# Severity assessment of accidents involving roadside trees based on occupant injury analysis

**DOI:** 10.1371/journal.pone.0231030

**Published:** 2020-04-07

**Authors:** Guozhu Cheng, Rui Cheng, Yulong Pei, Liang Xu, Weiwei Qi

**Affiliations:** 1 School of Traffic and Transportation, Northeast Forestry University, Harbin, Heilongjiang, China; 2 School of Civil Engineering, Changchun Institute of Technology, Changchun, Jilin, China; 3 Department of Civil Engineering and Transportation, South China University of Technology, Guangzhou, Guangdong, China; Tongii University, CHINA

## Abstract

The aims of this study were to achieve a quantitative assessment of the severity of accidents involving roadside trees on highways and to propose corresponding safety measures to reduce accident losses. This paper used the acceleration severity index (ASI), head injury criteria (HIC) and chest resultant acceleration (CRA) as indicators of occupant injuries and horizontal radii, vehicle departure speeds, tree diameters and roadside tree spacing as research variables to carry out bias collision tests between cars, trucks and trees by constructing a vehicle rigid body system and an occupant multibody system in PC-crash 10.0® simulation software. A total of 2,256 data points were collected. For straight and curved segments of highways, the occupant injury evaluation models of cars were fitted based on the CRA, and occupant injury evaluation models of trucks and cars were fitted based on the ASI. According to the Fisher optimal segmentation method, reasonable classification standards of severities of accidents involving roadside trees and the corresponding ASI and CRA thresholds were determined, and severity assessment methods for accidents involving roadside trees based on the CRA and ASI were provided. Additionally, a new index by which to evaluate the accuracy of the accident severity classification and the degree of misclassification was built and applied for the validity verification of the proposed severity assessment methods. A proportion of trucks was introduced to further improve the ASI evaluation model. For the same simulation conditions, the results show that driver chest injuries are more serious than driver head injuries and that the average ASI of cars is greater than that of trucks. The CRA and ASI have a positive linear correlation with the departure speed and a logarithmic correlation with the roadside tree diameters. The larger the spacing of roadside trees is and the smaller the horizontal radius is, the smaller the chance that a vehicle will experience a second collision and the lower the risk of occupant injury. In method validation, the evaluation results from two proposed severity assessment methods based on the CRA and ASI are consistent, and the degrees of misclassification are 4.65% and 4.26%, respectively, which verifies the accuracy of the methods proposed in this paper and confirms that the ASI can be employed as an effective index for evaluating occupant injuries in accidents involving roadside trees.

## Introduction

According to numerous accident investigations, a substantial number of collisions between vehicles and roadside trees are often caused by driver inattention or failures to make timely turns on curved segments of road. China's annual road traffic accident statistics [1] showed that accidents involving roadside trees accounted for 13.82% of single-vehicle accidents and that the maximum death rate of these accidents was 14.33% in 2018, which was higher than the death rate caused by collisions with other fixed objects, such as medians, guardrails and barriers. Trees with large diameters are commonly planted on roadsides, and fatalities can occur in collisions with these trees; thus, roadside trees have been regarded as key hazards.

To reduce losses due to roadside accidents, the “Roadside Design Guide 4th Edition” of AASHTO [2] proposed the concept of tolerant design. This concept suggests that an area where a driver who leaves the travel lane can regain control of their vehicle and safely return to the lane should be provided. Even if the errant vehicle cannot return to the lane, some degree of fault can be resolved. Therefore, fixed objects that hinder vehicle safety, including roadside trees, should not be located in this area. However, achieving a tolerant design due to the expensive cost of land is difficult. To reduce losses due to collisions with roadside fixtures, many researchers have performed optimizations of roadside fixture designs. For example, the instantaneous energy produced by a collision can be reduced by adopting breakaway supports for signs, poles, lighting and other roadside objects, which decrease the severity of accidents [3,4]; however, this optimization design does not apply to trees. Therefore, many researchers have proposed targeted improvement measures to mitigate losses in accidents involving roadside trees by exploring the relationship between roadside trees and roadside accidents.

In terms of the severity of accidents involving roadside trees, Daniello and Gabler [5] investigated 3,600 collisions between motor vehicles and roadside fixed objects in the US from 2004 to 2008 and believed that the death risk in collisions with roadside fixed objects was considerably higher than that in collisions with the ground, with the death rate of collisions with a guardrail and the death rate of collisions with trees being 7 times and 15 times, respectively, that of collisions with the ground. By collecting roadside accident data, Roque and Jalayer [6] revealed that the severity of collisions between vehicles and trees was twice that of collisions with other obstacles. Holdridge and Shankar et al. [7] employed the multinested logit model to confirm that planting thick and strong trees on the roadside tended to increase the probability of fatal roadside accidents. Schneider and Xie et al. [8,9] also verified that trees were identified as key injury risk factors in roadside accidents. Therefore, Ayati and Asghar Sadeghi et al. [10] considered trees as one of the risk indexes upon which to build a prediction model of roadside accident severity based on the evidential reasoning method. Wu et al. [11] incorporated a variety of variables (e.g., tree density, tree spacing and roadside depth) in a comprehensive evaluation index of roadside safety on highways and determined the threshold and standard of roadside safety evaluation based on classical set theory. Long et al. [12] constructed a tree model using LS-DYNA971 software and assessed roadside risks for secondary and tertiary highways in China via collision simulation tests. You et al. [13] incorporated the probability of vehicles driving out of the travel lane, the frequency of exposure to dangerous environments, and roadside hazards into an evaluation system for roadside risks. Roadside hazards included the offset distance and density of trees.

In terms of the number of roadside accidents, Lee and Mannering [14] investigated the relationship between roadside features and the frequency of roadside accidents and concluded that a decrease in the number of roadside trees can significantly reduce the occurrence of roadside accidents. Park and Abdel-Aty [15] concluded that an increase in the spacing of roadside trees can reduce the number of roadside accidents by establishing generalized nonlinear models and multivariate adaptive regression spline models. Jalayer and Zhou [4] believed that if warning signs or protective measures were installed around trees, poles and other dangerous objects, more than 38% of roadside accidents could be effectively reduced. In addition, Fitzpatrick and Harrington et al. [16] explored the influence of the roadside vegetation density on driver speed selection and vehicle lateral offset using simulation tests that aimed to explore the potential risks of roadside tree designs for driving safety.

Roque and Moura et al. [17] attempted to detect unforgiving roadside hazards that contribute to severe roadside accidents by evaluating driver injuries and the most severely injured occupants. Park and Abdel-Aty et al. [18] employed empirical Bayes and full Bayes methods to explore the influence of roadside barriers on roadside accident severities. Wu and Chen et al. [19] developed a mixed logit model to analyze severe driver-incapacitating injuries and fatalities in single-vehicle accidents on rural two-lane highways. Fang et al. [20] established a roadside environmental safety assessment model considering the two aspects of the possibility that a vehicle will leave the travel lane and the characteristics of roadside safety and divided roadside safety into five levels according to the comprehensive evaluation indexes of the model. Although numerous studies have investigated major roadside contributors to severe accidents, including poles, guardrails, bridge rails, ditches and barriers [21–31], these studies did not involve a risk indicator of roadside trees.

Most studies include trees as a risk factor in the research of roadside accidents and are limited to qualitative analyses. However, research on the severity evaluation of accidents involving roadside trees is lacking. The results of numerous roadside accident investigations and previous literature [7,15,32–34] concluded that vehicle speeds and roadside tree diameters are often related to the severity of roadside accidents and that the horizontal radii and spacing of roadside trees are often associated with the number of roadside accidents. However, the relationship between the horizontal radii, spacing of roadside trees and severity of roadside accidents remains unclear, and an explicit stipulation regarding the distance between roadside trees for safety in China has not been established; therefore, it is investigated in this paper. To realize the quantitative evaluation of the severity of accidents involving roadside trees, this paper utilizes PC-crash simulation software to build a car and truck rigid body system and an occupant multibody system. Given different departure speeds and road alignment conditions, collision tests between vehicles and trees with different diameters and spacings are conducted. In addition, the acceleration severity index (ASI), head injury criteria (HIC) and chest resultant acceleration (CRA) are considered alternative indexes by which to evaluate the injuries of occupants. The occupant injury evaluation models for trucks and cars are fitted for straight and curved segments of highways. The CRA and ASI thresholds are determined according to the Fisher optimal segmentation algorithm. Methods for evaluating the severity of accidents involving roadside trees based on the CRA and the ASI are proposed, and better verification is achieved using a newly proposed evaluation index (i.e., degree of misclassification). The research results of this paper can provide guidance and a reference for highway traffic management departments for promoting the implementation of more efficient speed limit countermeasures and carrying out the optimization design of roadside trees in the future.

## Methodology

### Occupant injury index

#### ASI

The ASI is the index proposed by the European Union (EU) Standard (1317-2-19-98) to evaluate occupant injury in collisions between vehicles and guardrails. The dimension of the ASI is 1, and the larger its value, the more serious is the occupant injury. When the ASI > 1, the crash intensity exceeds the limit that an occupant can endure. The calculation method is expressed as [35]:

$$\mathrm{ASI}\left(t\right)=\sqrt{{\left({\bar{a}}_{x}/{\hat{a}}_{x}\right)}^{2}+{\left({\bar{a}}_{y}/{\hat{a}}_{y}\right)}^{2}+{\left({\bar{a}}_{z}/{\hat{a}}_{z}\right)}^{2}}$$ (1)

where ${\hat{a}}_{x}$, ${\hat{a}}_{y}$ and ${\hat{a}}_{z}$ are the maximum bearing accelerations of occupants in the longitudinal direction, transverse direction and vertical direction, respectively, in cases in which occupants use seat belts; ${\hat{a}}_{x}$ is 12 *g*; ${\hat{a}}_{y}$ is 9 *g*; and ${\hat{a}}_{z}$ is 10 *g* (*g* = 9.8 m/s^2^). ${\bar{a}}_{x}$, ${\bar{a}}_{y}$ and ${\bar{a}}_{z}$ are the average maximum accelerations within 50 ms of the vehicle center of gravity position in the longitudinal direction, transverse direction and vertical direction, respectively, and can be obtained from the acceleration curve produced from collision tests after low-pass filtering. Their calculation formulas are expressed as follows [36]:

$${\bar{a}}_{x}=\frac{1}{\omega }\int_{t}^{t+\omega }a_{x}dt,\;{\bar{a}}_{y}=\frac{1}{\omega }\int_{t}^{t+\omega }a_{y}dt,\;{\bar{a}}_{z}=\frac{1}{\omega }\int_{t}^{t+\omega }a_{z}dt$$ (2)

where *a*_*x*_, *a*_*y*_ and *a*_*z*_ are the longitudinal acceleration, transverse acceleration and vertical acceleration, respectively, of the vehicle center of gravity position and *ω* is the time interval, which is 50 ms.

Although the ASI has been extensively employed in Europe, it primarily describes the acceleration characteristics of a vehicle in a collision with a guardrail and can only be used as an indirect index for evaluating occupant injuries. For other types of collision accidents, research that explores the relationship between the ASI and occupant injuries remains lacking. Therefore, this paper introduces commonly employed occupant injury indexes—the HIC and CRA—to further study the applicability of the ASI for occupant injury evaluation in accidents involving roadside trees by comparing the evaluation effects of these three indexes.

#### HIC

The HIC serves as the basis of the Federal Motor Vehicle Safety Standards (FMVSS), which are used to evaluate vehicle safety. In domestic and foreign laws and standards, the HIC has become the most extensively employed criterion for evaluating head injuries. The safety limit value for the HIC is generally 1,000. According to related statistics, when the HIC > 1,000, the probability of fatal head fracture will exceed 33%. The calculation formula for the HIC is expressed as [37]:

$$\mathrm{HIC}={\left[\left(t_{2}-t_{1}\right){\left(\frac{1}{t_{2}-t_{1}}\int_{t_{1}}^{t_{2}}a_{t}dt\right)}^{2.5}\right]}_{\max}$$ (3)

where *a*_*t*_ represents the resultant acceleration at the center of gravity of the head in a collision; *t*_1_ and *t*_2_ are two different times during the collision, which are chosen to maximize the HIC; and $\frac{1}{t_{2}-t_{1}}\int_{t_{1}}^{t_{2}}a_{t}dt$ represents the average resultant acceleration between *t*_1_ and *t*_2_. The time interval (i.e., from *t*_1_ to *t*_2_) of the HIC substantially affects the calculation of its value. Thus, this paper selects 15 ms, as adopted by the U-NCAP collision test.

#### CRA

The CRA is the resultant acceleration of the chest within the collision duration of 3 ms. The FMVSS state that the occupant's probability of survival is greatly reduced in a crash when the CRA > 60 *g*. Currently, the “Design Rules for Frontal Collision Occupant Protection (CMV-OR294)” of China also adopt this value as the safety limit.

### Fisher optimal segmentation methods

In this paper, the Fisher optimal segmentation method is used to classify the obtained test data to determine the classification scheme of the severity of accidents involving roadside trees, including the optimal classification number and the thresholds of injury indexes that correspond to each classification. The principle of this method is ensuring that the sum of the squares of deviation within each group of ordered sample data after segmentation is the minimum. At this point, the corresponding grouping situation is the optimal segmentation [38]. The following section discusses the implementation of optimal segmentation.

#### Define and calculate category diameters

The Fisher optimal segmentation method uses diameters to define differences between categories. The smaller the difference in a category is, the smaller the category diameter, which indicates that the sample attributes in this category tend to be consistent. If the data samples are divided into *k* categories, then $C_{m-1}^{k-1}$ classification methods exist. Assuming that *H*_*ij*_ is a certain category of $C_{m-1}^{k-1}$, the data samples in this category are denoted as {*x*_*i*_,*x*_*i*+1_,⋯,*x*_*j*_}(1 ≤ *i* < *j*). The sum of the deviation squares of *H*_*ij*_ is defined as the category diameter *D*(*i*, *j*) [39]:

$$D(i,j)=\sum_{i=1}^{j}{\left(x_{i}-{\bar{x}}_{ij}\right)}^{T}\left(x_{i}-{\bar{x}}_{ij}\right)$$ (4)

where *x*_*i*_ is the standardized sample value and ${\bar{x}}_{ij}$ is the mean value of the *i* sample to the *j* sample.

#### Calculate the classification error function

Generally, the classification error function is used to define the quality of the sample classification. If *m* data samples are divided into *k* categories, the corresponding classification error function is expressed as follows [40]:

$$e\left[p\left(m,k\right)\right]=\sum_{h=1}^{k}D\left(i_{h},i_{h+1}-1\right)$$ (5)

The error function of any classification method expresses the sum of various diameters; that is, the smaller *e*[*p*(*m*,*k*)] is, the smaller the sum of the diameters of all categories and the better the classification. The goal of the Fisher optimal segmentation method is to achieve the optimal segmentation of data samples by selecting a classification to ensure the minimum *e*[*p*(*m*,*k*)] and thus offer the best classification method.

#### Determine the optimal solution

According to formula (5), when *k* = 2, the classification error function that corresponds to the optimal two classifications is:

$$e\left[p\left(m,2\right)\right]=\underset{2\leq i\leq m}{\min}\left\{D\left(1,i-1\right)+D\left(i,m\right)\right\}$$ (6)

When k>2, the classification error function that corresponds to the optimal *k* classifications is:

$$e\left[p\left(m,k\right)\right]=\underset{2\leq i\leq m}{\min}\left\{e\left[p\left(i-1,k-1\right)\right]+D\left(i,m\right)\right\}$$ (7)

The appropriate classification point *i*_*k*_ needs to be determined to ensure that the calculated value of formula (7) is the minimum (i.e., retain *e*[*p*(*m*,*k*)] = *e*[*p*(*i*_*k*_−1,*k*−1)]+*D*(*i*_*k*_,*m*) as the minimum), and the *k* category sample *H*_*k*_ = {*i*_*k*_,*i*_*k*+1_,⋯,*m*} can be obtained. The classification point *i*_*k-*1_ is determined to satisfy *e*[*p*(*i*_*k*_−1,*k*−1)] = *e*[*p*(*i*_*k*-1_−1,*k*−2)]+*D*(*i*_*k*-1_,*i*_*k*_−1), and the *k*-1 category sample *H*_*k*-1_ = {*i*_*k*-1_,*i*_*k*-1_+1,⋯,*i*_*k*_−1} can be obtained. All classifications *H*_1_,*H*_2_,⋯,*H*_*k*_ can be generated by analogy, and the optimal solution can be determined.

#### Determine the optimal classification number

Generally, the optimal classification number is determined by drawing the curve of the minimum error function. When the curvature distinctly changes, the corresponding *k* is the appropriate classification number. To determine *k* more accurately, the ratios *β*(*k*) of the minimum error function between adjacent classification numbers should be calculated, as shown in formula (8). The optimal classification number *k* is further determined by comparing the ratio of the position where the curvature change is more distinct. The larger the ratio is, the better the classification effect.

β(k)=e[p(m,k)]/e[p(m,k+1)] (8)

### Degree of misclassification

To verify the accuracy of the proposed method for accident severity assessment in this paper, a new index—the degree of misclassification *α*—is established, which can accurately measure the degree of error in the classification of accident severity by the proposed method as:

$$\alpha =\frac{\sum_{i=1}^{n}\eta_{ij}FN_{ij}}{TN+\sum_{i=1}^{n}\eta_{ij}FN_{ij}}\;i\neq j$$ (9)

where *TN* is the number of correct classification cases; *FN*_*ij*_ is the number of cases in which the *i* occupant injury grade is misclassified into the *j* severity grade; *n* is the number of severity classifications; and *η*_*ij*_ is the corresponding weight coefficient, which can be calculated by $\eta_{ij}=\frac{\left|j-i\right|}{n}$.

## Simulation test

### Construct a test scenario

PC-crash 10.0®, which is a vehicle dynamics simulation software developed in Austria, is extensively applied in traffic accident modeling and crash testing. This paper uses this copyrighted software purchased by the School of Traffic and Transportation of Northeast Forestry University to carry out a simulation test. A two-way two-lane highway model with a lane width of 3.75 m and a hard shoulder width of 1.5 m is first established, then a tree rigid body model is employed as the roadside tree model, and the length, width and height of the tree rigid body model are adjusted to establish the road tree diameter and height. Since roadside trees in China are generally planted at the edge of the earth shoulder, roadside trees are placed 0.75 m from the edge of the hard shoulder in this test based on the provisions of the width of the earth shoulder in the “Design Specification for Highway Alignment (JTG D20-2017)” of China and the distance from the edge of the roadway for trees established in the “Technical Specifications for Planting and Maintenance Management of Street Trees (DB11/T 839–2017)” of China. Given the different adhesion performances of asphalt pavement and earth shoulder pavement, the adhesion coefficient of the driveway and hard shoulder is set to 0.7, and that of the earth shoulder is set to 0.5.

### Build the simulation model

In this paper, the BMW-116d Autom and Avia-A31 L-A31 are selected from the vehicle model database of PC-crash 10.0® as the representative models of cars and trucks, respectively. To study the injury degree of a driver in a collision, the multibody occupant system from the software is applied in the simulation test. By adjusting the attitude of the occupant, establishing the contact characteristics between the occupant and the seat and the connection characteristics between the seat and the vehicle body, and using the built-in spring damping element (equivalent to the safety belt) to restrain the occupant, the multibody system is designed.

Additionally, to define the contact characteristics between the occupant and the interior of the vehicle, the contact surface is drawn with the "Extrude" function of PC-crash 10.0® and then imported into the vehicle. According to our simulation experience, when the occupant multibody system is placed in the rigid body system of trucks, the simulation software presents extremely unstable collision characteristics. Therefore, this test only establishes the occupant multibody system in the BMW-116d Autom.

### Determine test parameters

Before collision testing, various test parameters must be determined. According to relevant provisions of the “Design Specification for Highway Alignment (JTG D20-2017)” of China and field investigation experience in accidents involving roadside trees, the values of the horizontal radius, vehicle departure speed, tree diameter, spacing of trees and vehicle type in this test are shown in Table 1.

**Table 1:** Test parameters.

Parameter	Value																					
**Horizontal radius (m)**	+∞		700			600			500			400			300			200			100	
**Vehicle departure speed (km/h)**	40			50			60				70		80				90			100		
**Tree diameter (cm)**	10	12			14	16		18		20		22		24		26		28	30			32
**Spacing of trees (m)**	2				3			4				5				6			7			
**Vehicle type**	“Truck” = 0											“Car” = 1										

Considering that the height of roadside trees has a certain impact on the severity of roadside accidents, the tree height should be established accordingly. According to the literature [41], the relationship between the height and diameter of roadside trees is expressed as:

$$H=32.7008e^{\frac{-8.7297}{D}}$$ (10)

where *H* is the height of roadside trees (m) and *D* is the diameter of roadside trees (cm).

Additionally, the angle of an errant vehicle that leaves the travel lane also affects the severity of roadside accidents. Many factors determine the angle. Since no relevant research is available in China, we refer to the statistics of roadside accidents of Europe in “Roadside Infrastructure for Safer European Roads” [42], in which there is a close relationship between the vehicle departure angles and the departure speeds in actual roadside accidents, as shown in Table 2.

**Table 2:** Relationship between the departure speeds and the departure angles.

Variable	Value						
**Departure Speed (km/h)**	40	50	60	70	80	90	100
**Departure Angle (°)**	12	11	9	9	8	7	6

### Test process

**Fig 1:**
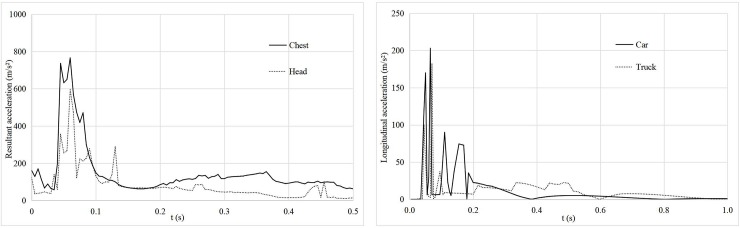
First collision type.

**Fig 2:**
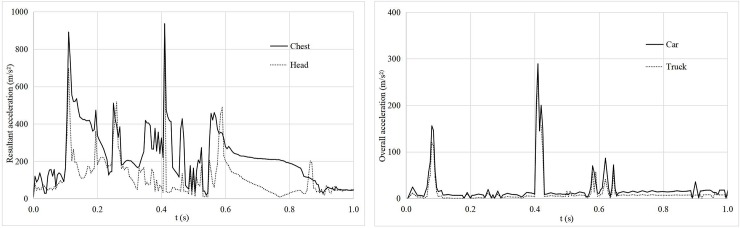
Second collision type.

**Fig 3:**
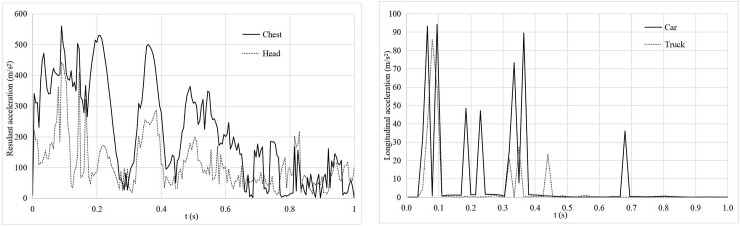
Third collision type.

By investigating numerous accidents involving roadside trees, it was determined that the collision positions of vehicles are primarily front middle and right-sided and that front right-sided collisions are more common. Therefore, offset collision testing involving collisions between front right-sided vehicles and roadside trees is performed in this study. The vehicle collision process can be presented vividly in three-dimensional form in PC-crash 10.0®. In addition, the longitudinal and lateral acceleration curves of the vehicles and the total acceleration curves of the vehicles, as well as the resultant acceleration curves of the driver's head and chest, can be obtained in this software, as shown in Figs 1–3. The CRA can be obtained, and the ASI and HIC can be calculated according to formulas (1) ~ (3).

Collision tests involving straight segments and curved segments are performed in this paper. In total, 282 straight segment data and 1,974 curved segment data, which yielded a total of 2,256 data, are collected. Based on the obtained test data, three common collisions with roadside trees are employed as examples to illustrate the acquisition method of the simulation data and explore the change rules of three occupant injury indexes for different collision types.

#### Vehicle rebounds after collision

In the section with a horizontal radius of 500 m, when vehicles with a speed of 80 km/h and a departure angle of 8° (as shown in Table 2) collide with a roadside tree with a diameter of 24 cm, height of 23 m (calculated by formula (1)) and a spacing of 4 m, the car and the truck do not cross the tree model after the collision but rebound to the left and rear. Their driving tracks comply with the rule of vehicle offset collisions. The acceleration curves of the vehicles are shown in Fig 1(A) and 1(B). According to Fig 1(A), the variation trends of the resultant acceleration of an occupant's chest and head are consistent. When t ≈ 0.06 s, the maximum resultant acceleration of the chest is 766 m/s^2^, the maximum resultant acceleration of the head is 596 m/s^2^, the corresponding CRA is 75 *g* > 60 *g*, and the HIC is 587 < 1,000. As shown in Fig 1(B), the longitudinal acceleration curves of the truck and car are the same. When the time t ≈ 0.06 s, the maximum longitudinal acceleration of the car is 203 m/s^2^, and the corresponding ASI is 1.77 > 1, while the maximum longitudinal acceleration of the truck is 182 m/s^2^, and the corresponding ASI is 1.73 > 1. According to this analysis, the degree of chest injury is greater than that of head injury for the car driver in the previous collision example. In addition to the HIC index, both the CRA index and the ASI index exceed the limits that occupants can bear.

#### Vehicle rotation after collision

When a vehicle collides with a tree at a speed of 90 km/h and a departure angle of 7°, keeping the other test parameters constant, the vehicle initially collides with the first tree and then rotates clockwise. The left side of the vehicle then contacts the adjacent second tree. The acceleration curves of the vehicle are shown in Fig 2(A) and 2(B). According to these two figures, when the time t ≈ 0.1 s and t ≈ 0.4 s, the resultant acceleration of the occupant and the total acceleration of the vehicle simultaneously reach the peak values, which correspond to the collisions between the vehicle and the first and second tree. The peak acceleration of the latter is greater than that of the former, which indicates that the secondary injury suffered by the occupant when the side of the vehicle collides with a roadside tree in the process of vehicle rotation is not less than the initial damage suffered by the occupant in the case of the vehicle front colliding with a roadside tree. When t ≈ 0.4 s, the maximum chest resultant acceleration of the occupant is 936 m/s^2^, and the corresponding CRA is 88 *g* > 60 *g*; the maximum head resultant acceleration is 768 m/s^2^, and the corresponding HIC is 743 < 1,000; the maximum total acceleration of the car is 289 m/s^2^, and the corresponding ASI is 2.12 > 1; and the maximum total acceleration of the truck is 273 m/s^2^, and the corresponding ASI is 2.07 > 1. Similarly, with the exception of the HIC index, both the CRA index and ASI index exceed the limits that the occupant can bear in the previously described collision.

#### Vehicle travels after collision

When vehicles with a speed of 60 km/h and a departure angle of 9° collide with a roadside tree with a diameter of 10 cm, a height of 14 m and a spacing of 3 m, keeping the other test parameters constant. After colliding with a roadside tree, vehicles pass through the tree model (equivalent to the vehicle breaking the tree) and then gradually stop moving. The acceleration curves of the vehicles are shown in Fig 3(A) and 3(B). According to Fig 3(A), during the entire collision process, the maximum resultant acceleration of the chest is 555 m/s^2^, the maximum resultant acceleration of the head is 441 m/s^2^, the corresponding CRA is 53 *g* < 60 *g*, and the HIC is 413 < 1,000. According to Fig 3(B), the maximum longitudinal acceleration of the car is 94 m/s^2^, and the corresponding ASI is 0.87 < 1, while the maximum longitudinal acceleration of the truck is 86 m/s^2^, and the corresponding ASI is 0.80 < 1. For this collision, three injury indexes are lower than the corresponding safety limit values, which indicates that the risk of occupant injury is lower when the vehicle collides with a small-diameter roadside tree.

## Results and discussion

### Variable analysis

#### Correlation analysis

According to the test data, the correlations between each test variable and occupant injury indexes are analyzed, as shown in Table 3, which shows that other occupant injury indexes are significantly correlated with each test variable, with the exception of the weak correlation between the HIC and the horizontal radius. Compared with the CRA and ASI, the HIC has a lower correlation level with each test variable. According to the analysis of the occupant injuries for the three collision types in the “Test process” section, compared with the CRA, the HIC seems to be unable to reflect the maximum degree of driver injury in cases of intense collisions. To confirm this finding, the relationships between the CRA, HIC and departure speed with the influences of other variables are explored using SPSS software in the next section.

**Table 3:** Correlation analysis.

	Departure speed	Horizontal radius	Tree diameter	Tree spacing	Vehicle type
**CRA**	0.942**	0.141**	0.537**	0.299**	--
**HIC**	0.678**	0.037	0.245**	0.116**	--
**ASI**	0.974**	0.071**	0.519**	0.380**	0.132**

** represents a significant correlation at the level of 0.01 (bilateral)

#### Relationship between the CRA, HIC and departure speed

**Fig 4:**
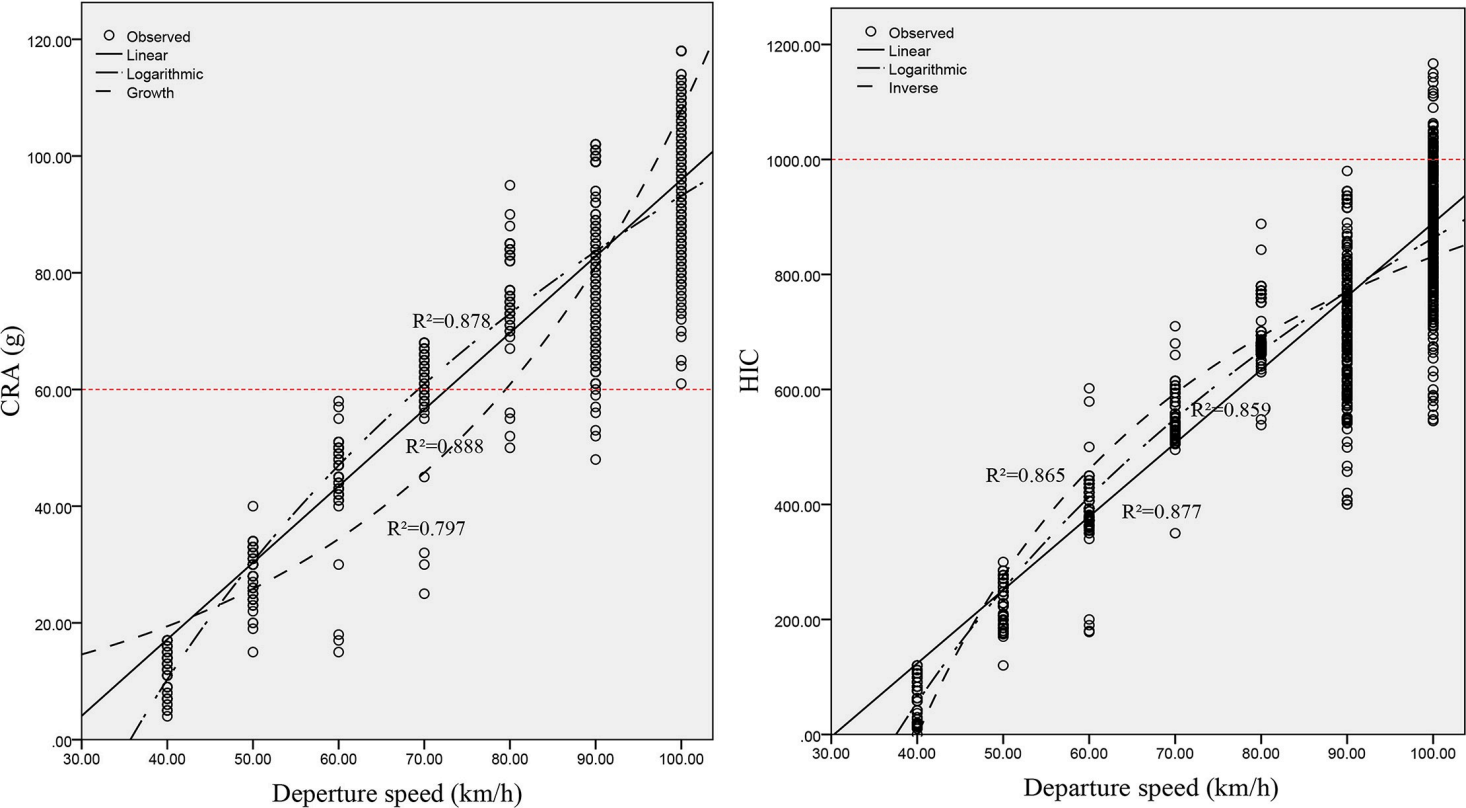
Relationship between the CRA, HIC and departure speed.

By comparing the correlation coefficients of different fitting curves, it is determined that the CRA and HIC have approximately positive linear relationships with the departure speed (as shown in Fig 4(A) and 4(B)). As shown in Fig 4, with an increase in the departure speed, the indicators of chest and head injuries gradually increase. With a departure speed ≥ 70 km/h, the CRA exceeds the safety limit value of 60 *g* (as shown in Fig 4(A)), and in the case of a departure speed ≥ 100 km/h, the HIC exceeds the safety limit value of 1,000 (as shown in Fig 4(B)). The results show that the chest injury is more serious than the head injury for the same collision type, which can be explained by the notion that the driver's chest is the first to contact the steering wheel in the car in the case of a front collision, which causes greater impact force on the chest and reduces the probability of serious head injury. Compared with the HIC, the CRA can more accurately evaluate the maximum degrees of injuries of occupants in this simulation test, and the evaluation effect is relatively ideal. In the following study, therefore, this paper abandons the HIC and selects the CRA and ASI to further evaluate and analyze the severity of accidents involving roadside trees.

To build a more accurate occupant injury assessment model, the relationships between the CRA, ASI and various test variables should be further explored.

#### Relationship between the CRA and the roadside tree diameter

**Fig 5:**
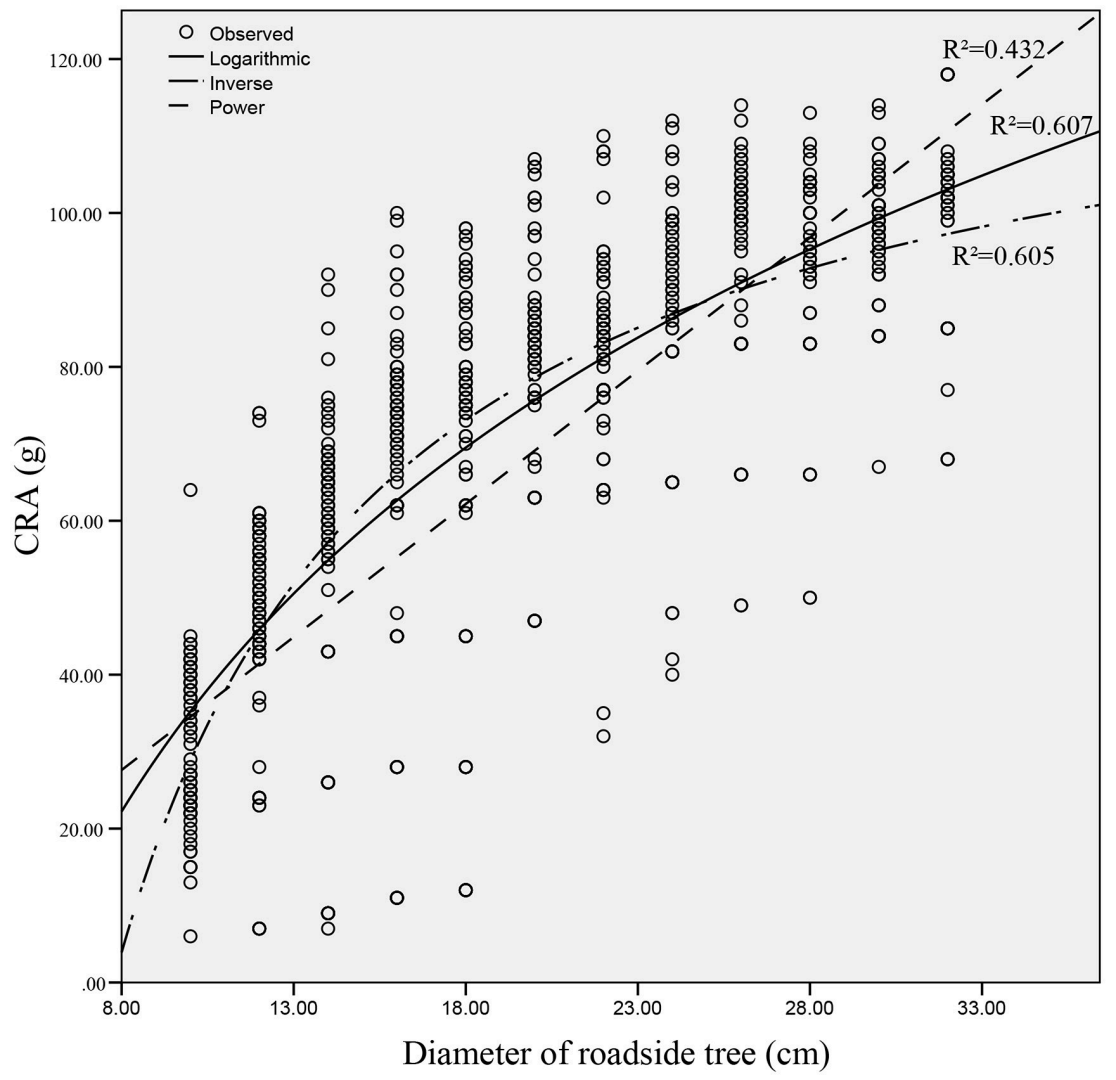
Relationship between the CRA and the diameter of roadside trees.

By comparing the correlation coefficients of different fitting curves, the relationship between the CRA and the diameter of roadside trees was determined to be approximately logarithmic (as shown in Fig 5). As the diameter of the roadside tree increases, the CRA gradually increases, but the increase continuously decreases.

#### Relationship between the ASI and the departure speed

**Fig 6:**
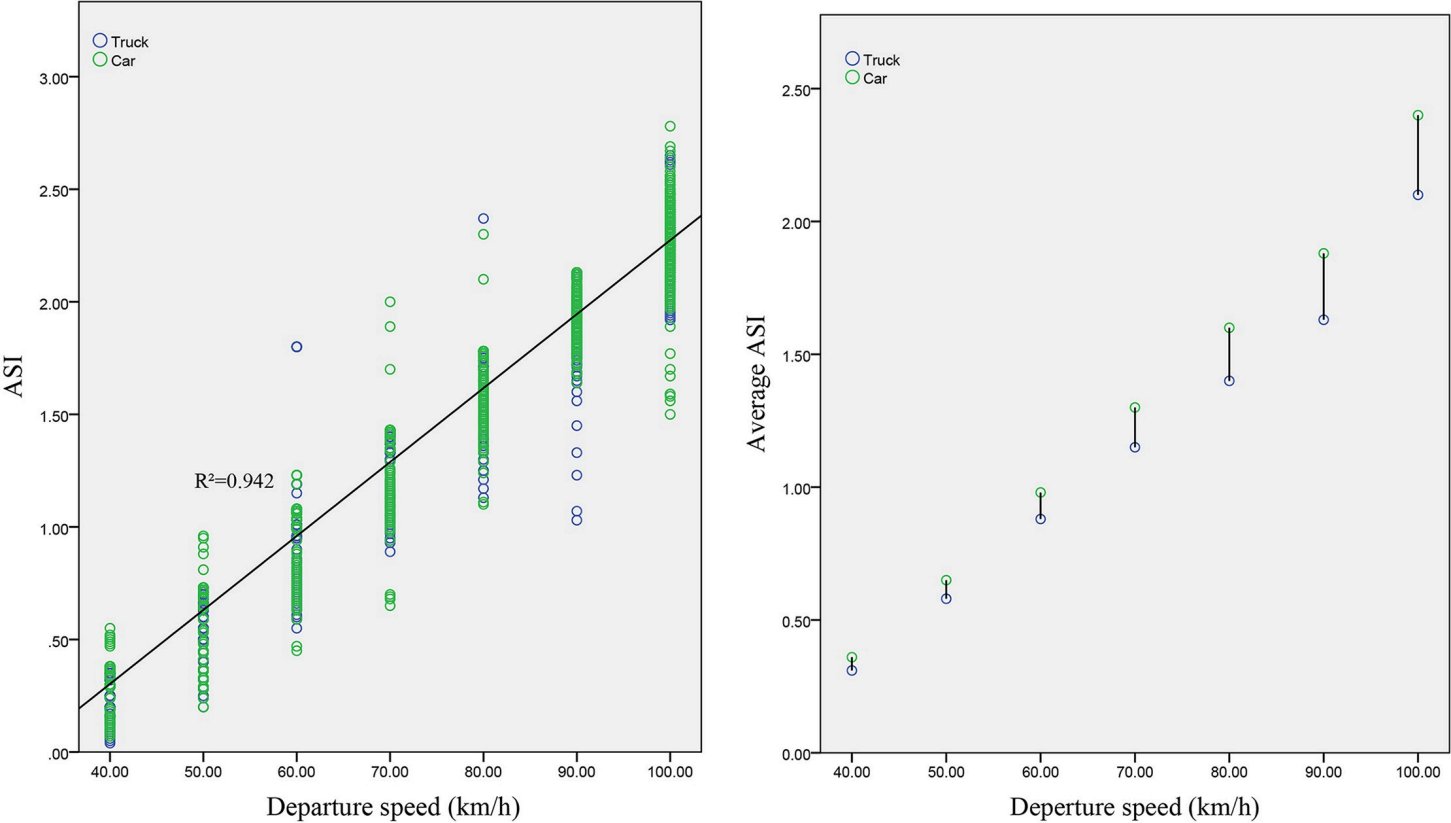
Relationship between the ASI and the departure speed.

An analysis of the relationship between the ASI and the departure speed (as shown in Fig 6(A)) concludes that the ASI is approximately and positively linearly correlated with the departure speed for both trucks and cars; that is, the ASI presents an increasing trend with an increase in the departure speed. To explore the differences between the ASI values of different vehicle types, Fig 6(B) shows the variation rule of the average ASI values of trucks and cars with the departure speed. The average ASI values of cars are greater than those of trucks, and the difference between the average ASI values of these two vehicle types gradually increases with increasing departure speeds.

#### Relationship between the ASI and the roadside tree diameter

**Fig 7:**
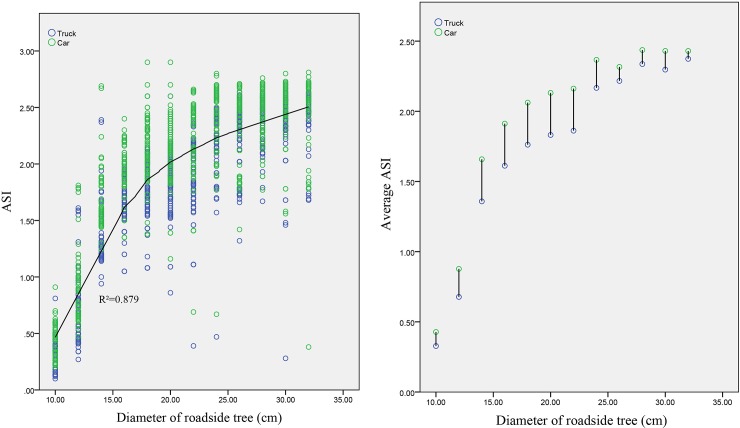
Relationship between the ASI and the tree diameter.

The relationship between the ASI and the diameters of roadside trees (as shown in Fig 7(A)) indicates that the ASI and the diameters of roadside trees are approximately logarithmically related for both trucks and cars. The ASI gradually increases with increasing diameters of roadside trees, and the increase continuously becomes smaller. Fig 7(B) shows the variation rule of the average ASI values of trucks and cars with the diameters of roadside trees. With an increase in the diameters of roadside trees, the difference in the average ASI value between these two vehicle types shows a trend of changing from small to large and then from large to small.

#### Relationship between the CRA, ASI, roadside tree spacing and horizontal radius

**Fig 8:**
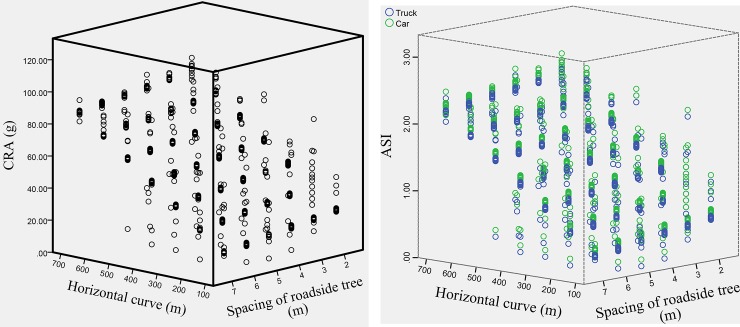
Relationship between the roadside tree spacing, horizontal radius and CRA as well as the ASI.

**Fig 9:**
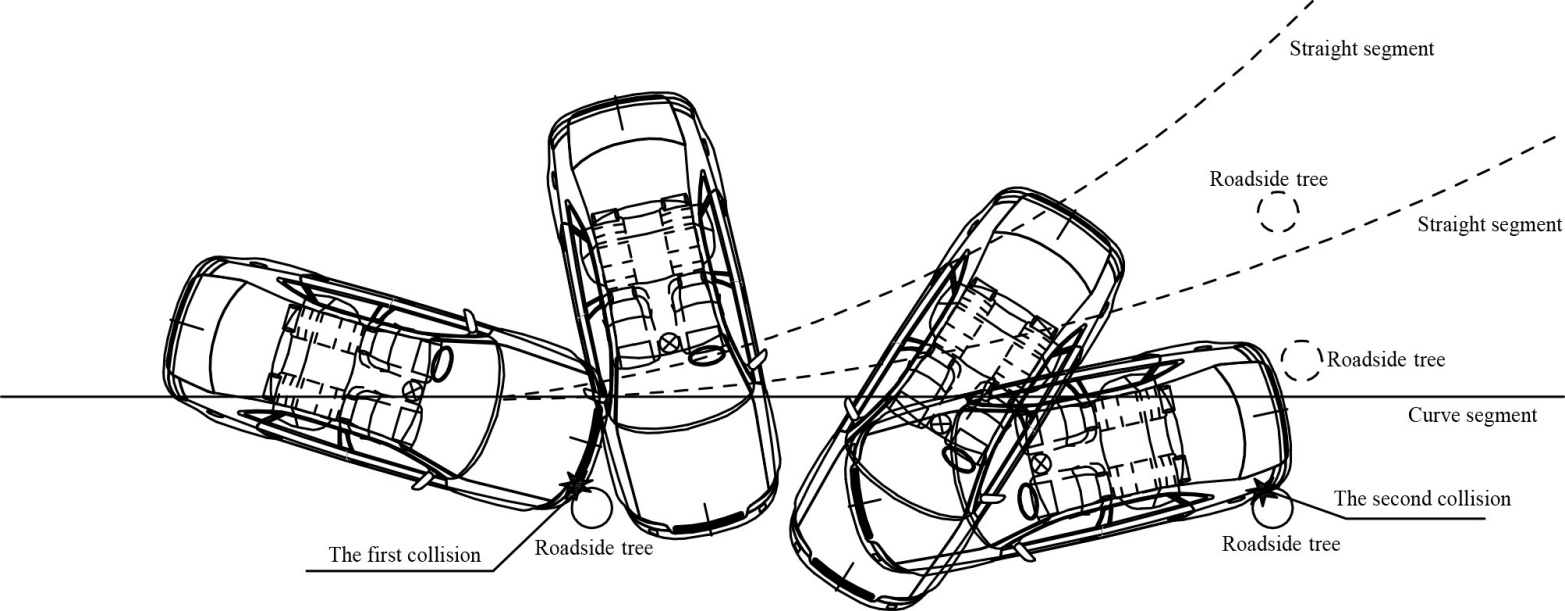
Schematic of the secondary collision.

To study the relationship between the roadside tree spacing, horizontal radius and CRA as well as the ASI, this paper employs the test data of the curve segment to draw three-dimensional graphs of these variables, as shown in Fig 8(A) and 8(B). Both the CRA and ASI show a decreasing trend with an increase in the roadside tree spacing and a decrease in the horizontal radius. The CRA and ASI are approximately inversely proportional to the roadside tree spacing and positively proportional to the horizontal radius. According to the combined experience of this simulation test, the larger the spacing of the roadside trees is, the smaller the horizontal radius and the smaller the probability of a second collision with adjacent roadside trees in the process of vehicle clockwise rotation after the first collision, which causes a lower risk of occupant injuries. The analysis principle is shown in Fig 9.

### Modeling

By exploring the relationship between the CRA, ASI and various test variables and taking into account the influence of the horizontal radius on the severity of accidents involving roadside trees as well as the difference between the truck ASI and the car ASI, SPSS software is used to conduct a regression analysis of the test data in this study. The occupant injury assessment models for cars based on the CRA and for both cars and trucks based on the ASI in straight and curved segments are fitted by comparing the correlation coefficients of the functions under different linear conditions, as shown in Table 4. The model expressions indicate that both the CRA and ASI have a multivariate nonlinear relationship with the departure speed, roadside tree diameters, roadside tree spacing and horizontal radii.

**Table 4:** Assessment models of occupant injuries.

No.	Model category	Road type	Model formula	R^2^
1	Assessment models of CRA	Straight segment	CRA = 1.417*V*_c_+8.1ln(*D*)−3.288*L*−57.099	0.925
2		Curve segment	$\mathrm{CRA}=1.382V_{c}+9.854\ln(D)-\frac{513.613L}{R}-63.195$	0.941
3	Assessment models of ASI	Straight segment	ASI_c_ = 0.035*V*_c_+0.233ln(*D*)−0.026*L*−1.726	0.988
4			ASI_t_ = 0.034*V*_t_+0.298ln(*D*)−0.014*L*−1.904	0.963
5		Straight segment	${\mathrm{ASI}}_{c}=0.035V_{c}+0.241\ln(D)-\frac{5.442L}{R}-1.751$	0.992
6			${\mathrm{ASI}}_{t}=0.034V_{t}+0.293\ln(D)-\frac{4.99L}{R}-1.884$	0.968

CRA refers to the resultant chest acceleration of car drivers (*g*).

ASI_c_ represents the acceleration severity index of cars.

ASI_t_ is the acceleration severity index of trucks.

*V*_c_ is the departure speed of cars (km/h).

*V*_t_ refers to the departure speed of trucks (km/h).

*D* is the diameter of roadside trees (cm).

*L* represents the spacing of roadside trees (m).

*R* is the horizontal radius (m).

### Accident severity classification

Based on the data obtained from the simulation test and combined with the CRA safety limit value introduced in the “Occupant injury index” section, the data of CRA ≤ 60 *g* are screened as a group, and the severity is set to level I. The remaining 757 groups of data are numbered in the order of the CRA values from small to large to generate CRA-ordered samples, which are denoted as *x*_*i*_ (*i* = 1,2,…,757). Similarly, according to the ASI safety limit value, the data of ASI_c_≤ 1 and ASI_t_ ≤ 1 are screened, and the severity is defined as level I. The remaining data are numbered according to the ASI value from small to large. ASI_c_-ordered samples are generated, which are denoted as *x'*_*i*_ (*i* = 1,2,…,866), and ASI_t_-ordered samples are generated, which are denoted as *x"*_*i*_ (*i* = 1,2,…,842).

**Fig 10:**
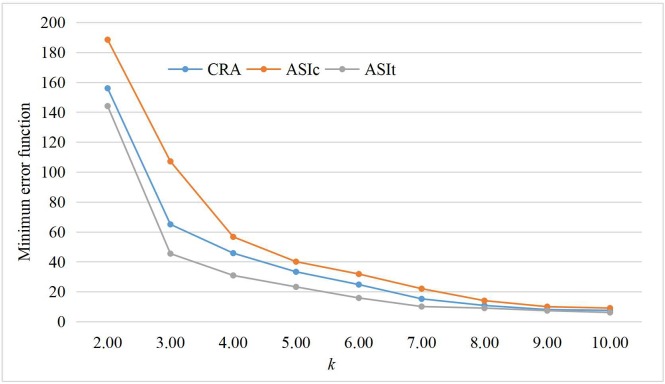
Relationship between the minimum error function and the classification number.

According to the defined ordered samples according to the CRA, ASI_c_ and ASI_t_, combined with the Fisher optimal segmentation method, this paper uses MATLAB software to achieve the optimal classification by writing the algorithm code. Based on the calculation of the category diameter and minimum error function of each ordered sample, this paper draws the curves of the minimum error function with different classification numbers *k*, as shown in Fig 10.

As shown in Fig 10, the minimum error functions of the three kinds of ordered samples show a monotonically decreasing trend with an increase in *k*. When *k* = 3 and 4, the curvature of these three kinds of curves significantly varies. To further determine the optimal classification number, the ratios of the minimum error function *β*(*k*) of the adjacent classification number are calculated in Table 5.

**Table 5:** Classification results.

Sample category	*k*	Minimum error function	Classification	*β*
CRA	2	156.005	{1~510} {511~757}	-
	3	65.023	{1~264} {265~510} {511~757}	1.42
	4	45.785	{1~264} {265~440} {441~510} {511~757}	1.37
	5	33.321	{1~187} {188~264} {264~440} {441~510} {511~757}	-
ASI_c_	2	188.453	{1~573} {574~866}	-
	3	107.125	{1~325} {326~573} {574~866}	1.89
	4	56.679	{1~325} {326~573} {574~779} {780~866}	1.41
	5	40.092	{1~163} {164~325} {326~573} {574~779} {780~866}	-
ASI_t_	2	144.095	{1~548} {549~842}	-
	3	45.462	{1~320} {321~548} {549~842}	1.47
	4	30.89	{1~320} {321~548} {549~738} {739~842}	1.33
	5	23.236	{1~131} {132~320} {321~548} {549~738} {739~842}	-

According to Table 5, for any ordered sample, the corresponding *β*(3) is greater than *β*(4). Thus, *k* = 3 is the optimal classification number for these three ordered samples. In addition, the codes that correspond to the optimal classification of each ordered sample can be obtained from Table 5, and then the corresponding injury index threshold can be determined. For example, the optimal classification for the CRA-ordered sample is {1~264} {265~510} {511~757}, and the corresponding injury index thresholds are (60 g, 73 g], (73 g, 96 g] and (96 g, +∞) according to the test data. Given that the severity of CRA ≤ 60 g is defined as level I and three threshold values obtained by the optimal classification correspond to level II, level III, and level IV, the threshold values of the CRA that correspond to different severity levels are shown in Table 6. The threshold values of the ASI_c_ and ASI_t_ can be obtained in a similar way.

Based on these research results and considering the classification of occupant injuries in traffic accidents in China, two classification assessment methods for the severity of accidents involving roadside trees based on the CRA and ASI are proposed, as shown in Table 6.

**Table 6:** Severity assessment methods for accidents involving roadside trees.

Severity grade	CRA threshold value	ASI_c_ threshold value	ASI_t_ threshold value	Occupant injury grade
I	≤60 *g*	≤1	≤1	Non- or minor injury
II	(60 *g*, 73 *g*]	(1, 1.78]	(1, 1.54]	Moderate injury
III	(73 *g*, 96 *g*]	(1.78, 2.21]	(1.54, 2.02]	Disabling injury
IV	>96 *g*	>2.21	>2.02	Fatal injury

### Method validation and improvement

To evaluate the accuracy of the classification evaluation methods for the severity of accidents involving roadside trees proposed in this paper, we explore the applicability of the ASI index in evaluating occupant injuries in accidents involving roadside trees. Fifty typical accident cases collected from the National Automobile Accident In-depth Investigation System (NAIS) in China were selected for verification, including 45 car accidents and 5 truck accidents, as shown in Table 7. First, according to the accident information, the corresponding CRA and ASI values of each accident are calculated by the occupant injury assessment models constructed in Table 4, and the corresponding accident severity grade is determined according to Table 6. The accident severity evaluation method is verified by the actual injuries of drivers in the accidents.

**Table 7:** Case validation.

No.	Accident speed (km/h)	Road type	Horizontal radius (m)	Roadside tree diameter (cm)	Roadside tree spacing (m)	Vehicle type	Driver injury grade	CRA	ASI	Severity grade
1	81	Straight	+∞	30	6	Car	Moderate	65 *g*	1.75	II
2	67	Straight	+∞	27	11	Truck	Moderate	--	1.21	II
3	95	Curve	3160	33	6	Car	Fatal	102 *g*	2.41	IV
4	60	Curve	2300	11	3	Car	Non	42 *g*	0.92	I
5	41	Straight	+∞	32	7	Car	Non	5 *g*	0.30	I
6	92	Straight	+∞	35	8	Car	Disabling	76 *g*	2.11	III
7	65	Curve	780	30	4	Car	Moderate	58 *g*	1.32	I/II^a^
8	76	Curve	2340	24	5	Car	Moderate	72 *g*	1.66	II
9	87	Curve	1540	25	8	Car	Disabling	86 *g*	2.04	III
10	93	Curve	2340	18	9	Car	Disabling	92 *g*	2.18	III
11	104	Curve	4560	22	10	Car	Fatal	110 *g*	2.62	IV
12	114	Curve	1260	28	12	Car	Fatal	122 *g*	2.99	IV
13	67	Curve	3410	34	4	Car	Moderate	64 *g*	1.44	II
14	89	Curve	560	15	3	Car	Disabling	84 *g*	1.99	III
15	76	Curve	270	30	5	Car	Moderate	66 *g*	1.63	II
16	56	Curve	1890	26	8	Car	Minor	44 *g*	0.97	I
17	81	Curve	690	25	5	Car	Disabling	77 *g*	1.82	III
18	69	Curve	1560	27	7	Car	Moderate	62 *g*	1.43	II
19	79	Curve	3570	33	5	Car	Disabling	80 *g*	1.85	III
20	78	Curve	2560	20	5	Car	Moderate	73 *g*	1.69	II
21	83	Curve	780	17	6	Car	Disabling	75 *g*	1.79	III
22	81	Curve	980	14	8	Car	Moderate	71 *g*	1.68	II
23	64	Curve	1320	10	7	Car	Minor	45 *g*	1.02	I/II^a^
24	61	Curve	2460	11	7	Car	Non	43 *g*	0.95	I
25	53	Curve	5270	18	5	Car	Non	38 *g*	0.80	I
26	77	Curve	670	30	4	Car	Disabling	74 *g*	1.73	III/II^a^
27	89	Curve	780	32	6	Car	Disabling	90 *g*	2.16	III
28	78	Curve	730	33	10	Car	Moderate	72 *g*	1.75	II
29	74	Curve	650	35	9	Car	Moderate	67 *g*	1.62	II
30	68	Curve	1780	25	8	Car	Moderate	62 *g*	1.38	II
31	69	Curve	1250	26	4	Car	Moderate	63 *g*	1.43	II
32	85	Curve	1200	22	6	Car	Disabling	82 *g*	1.94	III
33	90	Curve	500	24	2	Car	Disabling	90 *g*	2.14	III
34	97	Curve	610	20	7	Car	Fatal	101 *g*	2.30	IV
35	99	Straight	+∞	36	9	Car	Fatal	103 *g*	2.34	IV
36	67	Straight	+∞	12	8	Car	Fatal	32 *g*	0.99	I
37	56	Straight	+∞	15	11	Car	Non	8 *g*	0.58	I
38	101	Straight	+∞	40	5	Car	Fatal	99 *g*	2.54	IV
39	69	Straight	+∞	9	8	Car	Disabling	32 *g*	0.99	I
40	55	Straight	+∞	18	9	Car	Non	15 *g*	0.64	I
41	49	Straight	+∞	17	4	Car	Non	22 *g*	0.55	I
42	89	Straight	+∞	23	3	Car	Disabling	85 *g*	2.04	III
43	97	Straight	+∞	25	8	Car	Disabling	80 *g*	2.21	III
44	83	Straight	+∞	26	8	Car	Moderate	61 *g*	1.73	II
45	47	Straight	+∞	29	7	Car	Non	14 *g*	0.52	I
46	88	Straight	+∞	33	10	Car	Fatal	63 *g*	1.91	II/III^a^
47	86	Straight	+∞	32	5	Truck	Disabling	--	1.98	III
48	75	Straight	+∞	33	4	Truck	Disabling	--	1.63	III
49	70	Curve	560	31	6	Truck	Moderate	--	1.45	II
50	68	Curve	1230	27	7	Truck	Moderate	--	1.37	II

^a^The result of the severity grade before “/” is obtained from the severity assessment method based on the CRA, and that after “/” is obtained from the severity assessment method based on the ASI.

As demonstrated by the evaluation results in Table 7, the severity grades that correspond to 4 cases, cases 7, 36, 39 and 46, calculated by the severity assessment method based on the CRA are inconsistent with the drivers' injury grades in the accidents, with an error rate of 8.9%. The severity grades that correspond to 5 cases, cases 23, 26, 36, 39, and 46, calculated by the severity assessment method based on the ASI are inconsistent with the drivers' injury grades in the accidents, with an error rate of 10%. To further evaluate the degree of error classification of the proposed method, the degrees of misclassifications based on the CRA and ASI are calculated to be 4.65% and 4.26%, respectively, according to formula (9). Both values are within the acceptable range, thus verifying the accuracy of the classification assessment method for the severity of accidents involving roadside trees proposed in this paper. As shown in Table 7, in addition to cases 7, 23, 26 and 46, the evaluation results from these two methods for accident severity evaluation are consistent, which proves that the ASI index can also effectively evaluate the degree of occupant injuries in accidents involving roadside trees.

Considering the mix of cars and trucks on roads, this paper introduces the proportion of trucks *w* into models (3) ~ (6), as shown in Table 4. The improved ASI evaluation model is shown in models (11) and (12). If the proportion of trucks on the road section is known, a comprehensive evaluation of the severity of accidents involving roadside trees can be achieved.

1. Straight segment
  $$\begin{array}{l} \mathrm{ASI}=\left(1‐w\right){\mathrm{ASI}}_{c}+w{\mathrm{ASI}}_{t}\\ =\left(0.035-0.001w\right)V+\left(0.233+0.065w\right)\ln(D)\\ +\left(0.012w-0.026\right)L-\left(0.178w+1.726\right) \end{array}$$ (11)
2. Curved segment
  $$\begin{array}{l} \mathrm{ASI}=\left(1‐w\right){\mathrm{ASI}}_{c}+w{\mathrm{ASI}}_{t}\\ =\left(0.035-0.001w\right)V+\left(0.241+0.052w\right)\ln(D)\\ +\frac{\left(0.452w-5.442\right)L}{R}-\left(0.133w+1.751\right) \end{array}$$ (12)

where ASI is the vehicle acceleration severity index and *V* is vehicle departure speed (km/h). The corresponding improved ASI index thresholds are shown in Table 8.

**Table 8:** Improved ASI thresholds.

Severity grade	ASI threshold value
I	≤1
II	(1, 1.78–0.24*w*]
III	(1.78–0.24*w*, 2.21–0.19*w*]
IV	>2.21–0.19*w*

## Conclusion

In this study, the CRA, HIC and ASI are used as the occupant injury indexes; a vehicle rigid body system and an occupant multibody system are constructed in PC-crash simulation software; and offset collision tests involving cars, trucks and roadside trees are performed. Based on the analysis of the relationship between the test parameters and the occupant injury indexes, the occupant injury evaluation models of accidents involving roadside trees based on the CRA and ASI are fitted for straight segments and curved segments of highways, respectively. Upon defining CRA- and ASI-ordered samples, the Fisher optimal segmentation method is used to determine the reasonable evaluation grade of the severity of accidents involving roadside trees and the ASI and CRA thresholds at all grades. Assessment methods for the severity of accidents involving roadside trees based on the ASI and CRA are proposed to realize the quantitative evaluation of the severity of accidents involving roadside trees.

A new index for evaluating the accuracy of accident severity classification—that is, the degree of misclassification—is proposed. This index is applied in the case analysis to verify the effectiveness of the proposed accident severity assessment method in this paper and prove that the ASI can be used as an effective index by which to evaluate occupant injuries in accidents involving roadside trees.

The research results can provide guidance and references for highway traffic management departments for promoting the implementation of more efficient speed limit countermeasures and carrying out the optimization design of roadside trees as follows:

For a highway in operation, according to the horizontal radius, proportion of trucks, and diameters and spacing of roadside trees, the proposed severity assessment method in this paper can be used to calculate a speed value that can guarantee a severity grade ≤ II. The corresponding speed limit measures can be suggested in combination with the design speed of the road section.

For a highway in newly built or rebuilt areas, diameters and spacings of roadside trees with a severity grade ≤ II can be determined according to the horizontal radius, proportion of trucks and operating speed. Appropriate spacing can be selected for planting roadside trees according to the diameters of different varieties of mature trees.

In the investigation of actual roadside accidents, when the side slope is steep and the diameter of the roadside tree is small, errant vehicles tend to break the trees and then fall into the slope, which causes serious rollover accidents. To reduce the losses of this type of collision, a study of the optimal diameters of roadside trees can be carried out in the future. Roadside trees are regarded as a type of roadside protection facility, and as such, they can intercept a vehicle when struck by the vehicle via a buffering function to ensure minimum accident loss.
